# Precise Extrusion of Sweet Potato (*Ipomoea batatas* L.) Starch Sol Filaments: Printability Analysis and Process Optimization

**DOI:** 10.3390/foods15122204

**Published:** 2026-06-18

**Authors:** Al Kaxier G. Ancheta, Hiroyuki Kozu, Takumi Umeda, Marcos A. Neves, Isao Kobayashi

**Affiliations:** 1 Graduate School of Science and Technology, University of Tsukuba, Tsukuba 305-8577, Japan; agancheta2@up.edu.ph; 2 Department of Engineering Science, College of Engineering and Agro-industrial Technology, University of the Philippines Los Baños, Los Baños 4031, Philippines; 3 Institute of Food Research, National Agriculture and Food Research Organization, Tsukuba 305-8642, Japan; kozu.hiroyuki484@naro.go.jp (H.K.); umeda.takumi880@naro.go.jp (T.U.); 4 Institute of Life and Environmental Sciences, University of Tsukuba, Tsukuba 305-8577, Japan; marcos.neves.ga@u.tsukuba.ac.jp; 5 School of Integrative and Global Majors, University of Tsukuba, Tsukuba 305-8577, Japan

**Keywords:** sweet potato starch, sol, filament, precise extrusion, 3D food printing

## Abstract

Three-dimensional (3D) food printing, a relatively new food-processing method, was explored using gelatinized sweet potato starch (SPS) as a food ink. Prior to the production of intricate 3D shapes, this study focused on the precise extrusion of filaments, specifically the optimization of the printing conditions for nozzle diameters of 1.5 and 4.0 mm to produce filaments with an acceptable appearance and size. The rheological and mechanical properties of the (SPS) sol were also determined to describe the extrudability and shape retention of the food materials. The optimization process employed the Response Surface Methodology (RSM) and a desirability function to generate mathematical models of the width and height of the filaments as functions of the moisture content, the print temperature, and the print speed. The generated mathematical models were used to determine the optimum printing conditions. Hence, for the 1.5 mm nozzle, the optimum condition was at 82% moisture content, 57 °C print temperature, and 10 mm/s print speed, with a desirability of 0.842. In contrast, for the 4.0 mm nozzle, the optimum condition was at 82.3% moisture content, 50 °C print temperature, and 5 mm/s print speed, with a desirability of 0.911. The optimized filaments are expected to be used in 3D food printing to create 3D shapes.

## 1. Introduction

Three-dimensional (3D) food printing is a relatively new technology that can produce intricate food structures with high precision. In this paper, extrusion-based 3D food printing was employed wherein effective shape formation relies on precise extrusion and stable filament formation. The food material is forced through a nozzle to produce filaments that ideally match the nozzle geometry. However, the filament dimensions often deviate from the nozzle dimensions because of the rheological and mechanical properties of the material and the printing conditions. Such deviations influence the dimensional accuracy, appearance, and structural stability of 3D-printed objects.

Successful 3D food printing strongly depends on the selection of appropriate food materials as food inks, such that the food material should be extrudable and able to maintain its shape after extrusion. Among the potential food inks, starch, one of which is sweet potato starch (SPS), is highly suitable for 3D food printing because its gelatinized form exhibits shear-thinning behavior, viscoelasticity, and ease of deformation under shear stress [1].

SPS is an abundant and sustainable raw material. It accounts for approximately 50–80% of the dry weight of sweet potatoes [2]. In 2022, global sweet potato production reached approximately 86.4 million metric tons [3]. The starch typically contains 20–30% amylose and 70–80% amylopectin, enabling the formation of highly viscous pastes during heating and strong gels upon cooling. Since this amylose-to-amylopectin ratio is representative of many native starches, SPS also serves as a useful model starch for investigation.

Previous studies have investigated modifications of SPS to improve its gelling properties [4,5]. However, the present study focused on the simplest system consisting only of native starch and water, such that addition of food additives and physical or chemical starch modification were not yet considered. The preliminary experiments using starch dough, composed of native and partially gelatinized starch, exhibited shear-thickening behavior, which is undesirable for extrusion-based printing [6,7]. In contrast, fully gelatinized starch displayed shear-thinning behavior, facilitating extrusion and promoting shape retention after deposition, which are considered favorable for 3D food printing [8].

In this study, SPS was prepared as a sol, defined as a hot colloidal mixture of starch and water produced during gelatinization. This differs from a starch gel, which forms upon cooling when water becomes entrapped within a three-dimensional starch network. SPS sol has several potential applications in 3D food printing. The extruded filaments may be used to produce starch-based noodles as an alternative to conventional extrusion processes [6,9,10,11]. The material may also serve as a carrier for functional ingredients such as probiotics [12], anthocyanins [13], and polyphenols [14]. Furthermore, additives including agar, konjac, guar gum, locust bean gum, xanthan gum, sodium alginate, and pectin may be incorporated to enhance printability [15,16,17,18,19], while the gel form of the 3D-printed starch has potential applications in topical drug delivery systems [20].

A key term in 3D food printing, printability describes the ability of a food material to be extruded through a nozzle to form continuous filaments that reproduce a predefined shape and retain their structure after deposition. Though there are many ways to evaluate printability, it may be assessed by comparing the intended dimensions of a printed structure with the obtained dimensions [21]. In this study, the printability was judged based on the rheological and mechanical properties of the starch sol, together with the width and height of the extruded filaments compared to the nozzle diameter. Although the printability of several starch-based materials, including agar–konjac systems [15], radio-frequency-modified potato starch [22], pumpkin paste [23], and nixtamalized corn [24], have been investigated, studies specifically examining SPS sol are still limited [25,26].

Since SPS sol has relatively high viscosity and mechanical strength, syringe-based extrusion was selected instead of screw-based extrusion to produce high-resolution structures made of filaments [27]. Filament quality is influenced by several processing variables, including moisture content, print temperature, extrusion speed, and print speed. For instance, the print temperature must remain above the gelation temperature for a filament, which is a viscoelastic material, to maintain a fluid-like behavior during extrusion, after which the deposited filament cools and becomes sufficiently solid-like to retain its shape. In addition, a smooth and continuous filament is generally achieved when the ratio of extrusion speed to print speed (V/U) approaches unity [28].

Therefore, the objective of this study was to systematically investigate the effects of moisture content, print temperature, and print speed on the extrusion behavior of SPS sol using nozzle diameters of 1.5 and 4.0 mm. The study also aimed to optimize these printing conditions to achieve precise filament extrusion, such that the filament width and height would closely match the corresponding nozzle diameters.

## 2. Materials and Methods

### 2.1. Source of Sweet Potato Starch

SPS was purchased from Nao Bussan Co., Ltd. (Osaka City, Japan). The sample was stored in a relative humidity (RH) chamber maintained at approximately 10% RH prior to experimentation. Prior to use, the starch was allowed to equilibrate with ambient air for approximately 30 min to set its initial moisture content, before adding a calculated amount of water to establish the moisture content of the starch sol for use in precise extrusion.

### 2.2. Preparation of Sweet Potato Starch Sol

Before starch sol preparation, the moisture content of the SPS was determined using a moisture analyzer (MS-70, A&D Co., Ltd., Tokyo, Japan). This was performed to calculate the amount of water added to the native starch to achieve a certain moisture content prior to heating.

SPS sol was prepared by mixing native SPS and Milli-Q^®^ ultrapure water in a beaker to form a slurry with a specific moisture content (MC). The slurry was then heated in a water bath at 90 °C for approximately 1–2 h or until full gelatinization (i.e., homogeneous and translucent). Subsequently, the temperature of the gelatinized starch was maintained at 50–70 °C (depending on the print temperature setting of the 3D food printer) in the same water bath to prevent gelation. The beaker was covered with aluminum foil to prevent moisture loss prior to loading it into the capsule body of the 3D food printer.

### 2.3. Rheological Properties Determination

The rheological properties of SPS sol were determined using a rheometer (HAAKE MARS iQ Air, Thermo Fisher Scientific Inc., Waltham, MA, USA). Measurements were obtained at 50, 60, and 70 °C for freshly prepared samples with 82%, 87%, and 92% moisture content (adopting the same printing conditions during precise extrusion) using a 35 mm diameter flat plate with a 1 mm gap between the plate and the sample stand. Prior to the analysis, the sol samples were immersed in a water bath at the same temperature as that of the rheometer to prevent gelation. Three modes were performed using the rheometer: flow curve, strain sweep, and stress sweep, and several graphs were generated. The range of shear rate values was 0.01–100 s^−1^. The frequency was set at 1 Hz. The shear stress range was 1–1000 Pa. The Herschel–Bulkley parameters were also calculated. Triplicate samples were prepared during the analysis to check the repeatability.

### 2.4. Texture Profile Analysis

The mechanical properties of the samples were analyzed using a texture meter (TPU-2, Yamaden Co., Ltd., Tokyo, Japan). Before the analysis, the sol samples were immersed in a water bath at the desired temperature (50, 60, or 70 °C), given that the texture meter had no temperature control system. Approximately 28 g of each freshly prepared sample (as in Section 2.3) was placed in a stainless-steel container with a diameter of 48 mm and a height of 15 mm. The filled container was then placed in a texture meter for texture profile analysis (TPA). The compression process was performed using a 16-mm diameter plunger. The other texture meter settings were a compression speed of 5 mm/s, a clearance of 5 mm, and two measurement cycles. Hardness, adhesiveness, and cohesiveness were measured using TPA curves. In addition, the Young’s modulus was calculated as the slope of the elastic region of the stress–strain curves generated by the same equipment at strain values of 4–10%, where the linearity was the highest. Starch sol samples were analyzed immediately after preparation. Triplicate samples were prepared for use in the texture meter.

### 2.5. Precise Extrusion

**Figure 1:**
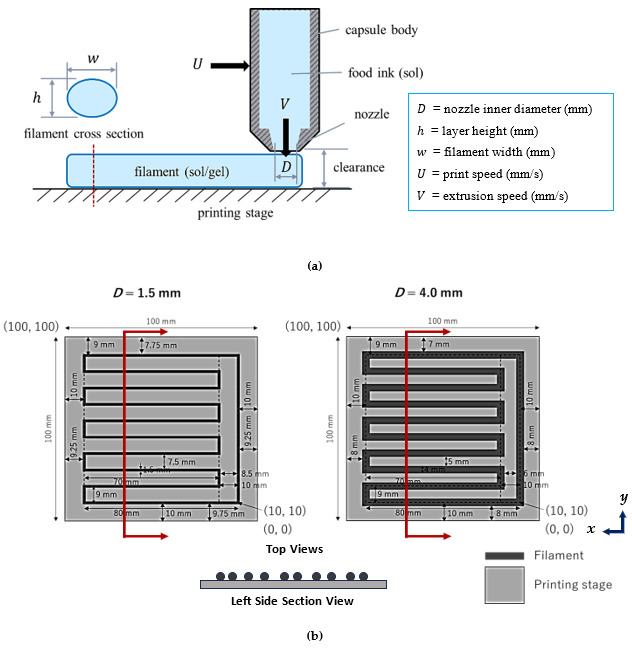
Printing setup for the precise extrusion of sweet potato starch (SPS) sol filaments, showing (**a**) the equipment setup and (**b**) the line pattern of the filaments. (Red cutting plane lines indicate the direction of cutting and and the exposed section, hence, the left side section view).

SPSsol filaments were extruded using a syringe-type 3D food printer (Foodini^®^, Natural Machines, Barcelona, Spain). Approximately 100 g of freshly prepared starch sol per batch was loaded into the capsule body prior to extrusion. The setup of the equipment used for precise extrusion is shown in Figure 1a.

The range of moisture content values of 82–92% was determined based on a preliminary experiment, such that below the lower limit (82%), the printer could hardly extrude the food material using its piston, and above the upper limit (92%), the food material would flow out of the nozzle by gravity alone.

The printer settings were determined based on preliminary experiments. Nozzle diameters of 1.5 and 4.0 mm were used, as they were available in the laboratory. Print speeds of 10, 25, and 40 mm/s for the 1.5 mm nozzle and 5, 52.5, and 100 mm/s for the 4.0 mm nozzle were carefully chosen. For the print temperature, 50, 60, and 70 °C were used, and a waiting time of approximately 30 min was allotted for the sample inside the capsule to adapt to the set temperature before extrusion. Prior to the precise extrusion operation, the capsule was attached to a heating system with a thermostat to control the temperature and was released when the operation started. The appropriate clearance between the tip of the nozzle and the printing stage was also established depending on the nozzle diameter (2.5 mm for the 1.5 mm nozzle and 5.5 mm for the 4.0 mm nozzle). Other settings were an ingredient flow speed of 1.7 and a turning speed factor of 1 (“1” for elastic materials and “0” otherwise).

### 2.6. Printing Pattern of the Sweet Potato Starch Sol Filaments

The printing (line) pattern of the filament on the printing stage was designed such that ten filaments could be generated for dimensional measurements, as shown in Figure 1b. Based on the orientation of the x- and y-axes, the printing started at (10, 10), moved to the left to print the first horizontal filament, then moved upward to print the nine remaining filaments, and moved downward and ended at the starting point. After printing, the filaments were allowed to cool to room temperature for approximately 5 min before being cut to expose the ten cross sections that were analyzed for width and height.

### 2.7. Measurement of Mass and Dimensions

The mass of the samples was measured using a balance to determine the total mass of all ten filaments per stage. The width and height of the filaments were measured using a caliper (Absolute Digimatic^®^, Mitsutoyo, Co., Kawasaki, Japan) within 30 min of printing. Area, contact angle, and eccentricity were determined using ImageJ^®^ software (ImageJ 1.46r, National Institutes of Health, Bethesda, MD, USA) based on photographs of the left section view of the filaments using a camera (Cyber-shot DSC-HX99, DSC-HX90V, Sony Co., Tokyo, Japan). The mean width and height were calculated as the average of ten filaments.

### 2.8. Design of Experiment (DOE) and Statistical Analysis

Table 1 shows the three-factor experimental design for optimizing the precise extrusion of sweet potato starch sol using 1.5 and 4.0 mm nozzles.

The experimental design was based on a complete factorial design (CFD) with three factors (moisture content, print temperature, and print speed) and three levels for each factor. Two responses were obtained: width and height. Based on the number of factors and levels (three factors for each response and three levels for each factor), there were 3^3^ = 27 runs plus one additional run for the center point (all middle values of moisture content, print temperature, and print speed) for a total of 28 runs for each nozzle diameter. Three levels of each factor were considered to enable detection of nonlinear (i.e., quadratic) effects where the response did not necessarily change linearly given the factors. In addition, the software used for optimization suggested two center points to produce more valid mathematical models. Response Surface Methodology (RSM) and a desirability function were applied to the experimental data to generate regression models and calculate the desirability values while performing ANOVA for Model (calculation of p-value of each term in a model and p-value of the whole model equation) and analysi of variance (ANOVA) for Residual (p-value from lack of fit and pure error). The optimum moisture content, print temperature, and print speed were calculated using the Stat-Ease^®^ 360 software (trial version, Stat-Ease, Inc., Minneapolis, MN, USA, www.statease.com) for nozzle diameters of 1.5 mm and 4.0 mm. Subsequently, the model width and height values corresponding to the optimum conditions were compared with the experimental width and height values based on the calculated optimum conditions.

The determined values are expressed as the mean ± standard deviation. Data were analyzed using ANOVA, followed by Tukey’s honest significant difference (HSD) test at a significance level of 5%.

## 3. Results and Discussion

### 3.1. Rheological Properties

**Figure 2:**
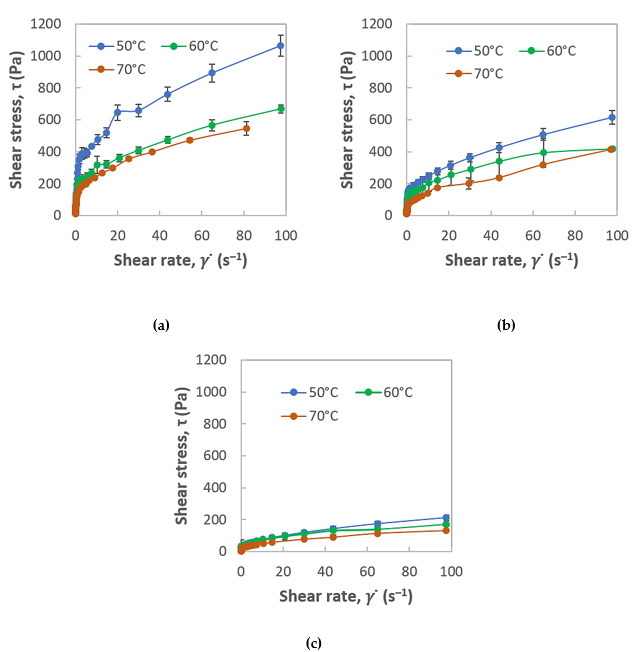
Flow curves of sweet potato starch (SPS) sol at (**a**) 82%, (**b**) 87%, and (**c**) 92% moisture contents.

Figure 2 shows the flow curves (shear stress versus shear rate) for SPSsols with different moisture contents and print temperatures. The corresponding apparent viscosities are shown in Figure S1 in the Supplementary Material.

As shown in the flow curves (Figure 2), the shear stress increased with the shear rate; however, the slope of the shear stress–shear rate curve decreased at higher shear rates, indicating shear-thinning behavior of the food material, which is desirable for precise extrusion. In addition, lower moisture content and print temperature tended to make the deformation (in terms of shear rate) more difficult because of the higher shear stress required to cause the deformation. A higher moisture content and print temperature caused the food material to be less viscous and flow more easily (see Figure S1 in the Supplementary Material) during extrusion.

**Figure 3:**
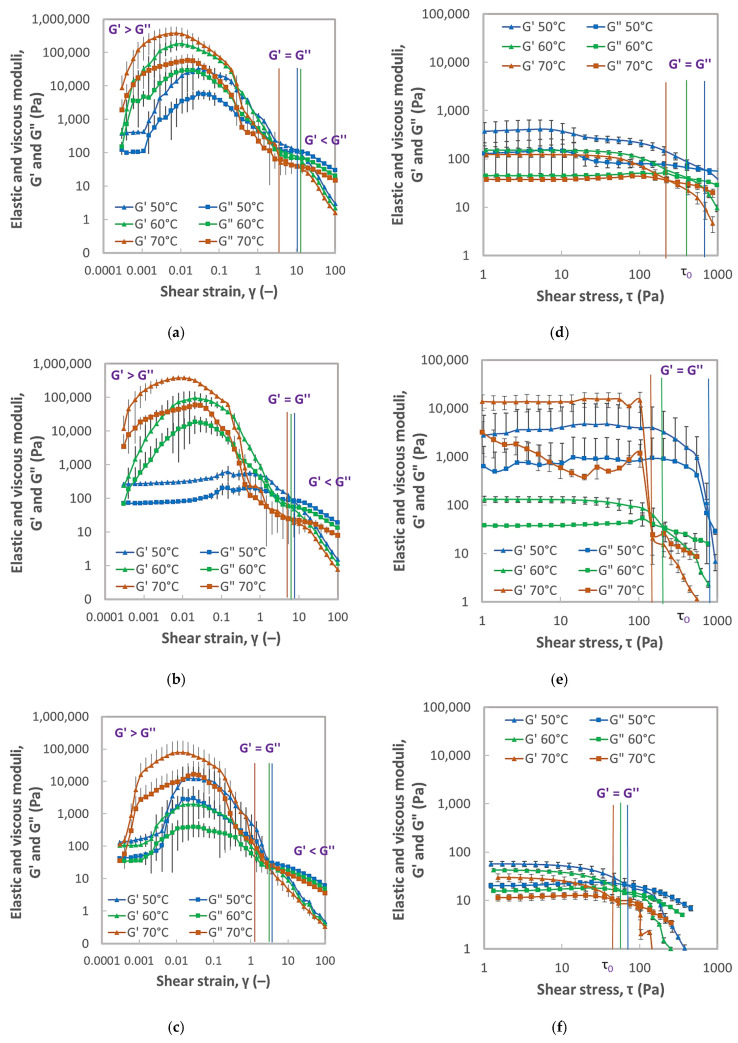
Elastic modulus ($G^{\prime }$) and viscous modulus ($G^{\prime \prime }$) for sweet potato starch (SPS) sol as affected by shear strain (γ) at (**a**) 82% moisture content, (**b**) 87% moisture content, and (**c**) 92% moisture content; and by shear stress (τ) at (**d**) 82% moisture content, (**e**) 87% moisture content, and (**f**) 92% moisture content. (The frequency of the rheometer is 1 Hz. Vertical lines indicate the locations of the crossovers of G′ and G″).

In Figure 3, the elastic modulus ($G^{\prime }$) and viscous modulus ($G^{\prime \prime }$) are plotted against the shear strain (a, b, and c) and the shear stress (d, e, and f), respectively, for the SPS sols. Shear strain pertains to the angular deformation of the food material when shear stress is applied, whereas shear stress is the force divided by the area parallel to the force, resulting in deformation of the material by sliding or rotating. The crossovers of $G^{\prime }$ and $G^{\prime \prime }$ indicate a transition of the viscoelastic food material from more solid-like ($G^{\prime }>G^{\prime \prime }$) to more fluid-like ($G^{\prime }<G^{\prime \prime }$) when the strain is increased.

Before the crossover, or at very low values of shear strain, higher values of $G^{\prime }$ compared to $G^{\prime \prime }$ suggest that the food material has a high degree of elasticity, which is favorable after extrusion because the material has the ability to retain its shape. However, at higher strain values, the higher values of $G^{\prime \prime }$ compared to $G^{\prime }$ suggest that the material can deform permanently when shear stress is applied, which is advantageous for extrusion.

Figure 3d–f were used to determine the yield stress of the food material at varying moisture contents and print temperatures by projecting the crossover of $G^{\prime }$ and $G^{\prime \prime }$ (or yield point) onto the horizontal axis. The yield stress is the shear stress at which the material begins to flow and deforms continuously. During extrusion, the yield stress must be exceeded such that the viscoelastic material behaves more similar to a liquid, thus facilitating the extrusion process; in particular, the shape and size of the nozzle and the flow through the nozzle and on top of the printing stage are important factors.

The effects of the moisture content and the print temperature on the values of $G^{\prime }$ and $G^{\prime \prime }$ were observed. Generally, increasing the moisture content and the temperature resulted in lower values of $G^{\prime }$ and $G^{\prime \prime }$. However, for SPS sols, the moisture content and the print temperature may have a synergistic effect on the interaction between the water and starch molecules, which influences the resistance of the viscoelastic material to deformation. This synergistic effect was reflected in the width and height of the extruded filaments, which were affected by the rheology of the food material, as discussed in the following sections.

The Herschel–Bulkley model was used to calculate the consistency coefficient (K) and the flow index (n) given the yield stress ($\tau_{0}$) that was determined from Figure 3d–f.

(1) $$\tau =\tau_{0}+K{\dot{\gamma }}^{n}$$

The values of $\tau_{0}$, K, and n are summarized in Table 2.

Yield stress is an important parameter because it measures the strength of the food material structure during extrusion. A higher $\tau_{0}$ means more stress is needed to initiate the flow of the material during extrusion. At 82% and 87% moisture contents, the $\tau_{0}$ slightly decreased when the print temperature increased. However, this change was not significant. At 50 °C and 60 °C print temperatures, the $\tau_{0}$ somewhat decreased when the moisture content increased. However, at 70 °C, the change in the $\tau_{0}$ due to the moisture content was no longer significant. Nevertheless, increasing the moisture content and the print temperature resulted in a lower yield stress. On the other hand, a higher *K* means a higher apparent viscosity and a greater shear stress are needed to cause deformation (as shear rate) of the food material; thus, there is greater difficulty extruding the food material. A similar trend was found in the values of K such that it can be lowered by increasing the moisture content and the print temperature. Finally, for n, no significant difference was found between the data points. Since all the values of n (0.55–0.79) were between zero and one, the shear-thinning behavior of the food material was verified and was desirable for precise extrusion.

### 3.2. Mechanical Properties

The mechanical properties of the SPS sol, such as hardness, cohesiveness, adhesiveness, and Young’s modulus, are listed in Table 3.

The hardness was significantly higher (p< 0.05) at 82% moisture content. However, the print temperature had no significant effect on the hardness. The hardness of a food material is important because it indicates its ability to resist deformation. This explains why the extruded filaments with lower moisture contents exhibited better shape retention after printing. Compared to other food materials, such as nixtamalized corn with values in the range of 5260–112,230 Pa [24] and pumpkin paste with values in the range of 10,160–141,560 Pa [23], SPS sol is a relatively soft food material with a hardness in the range of 718–2911 Pa at varying moisture levels and print temperatures. The softness of the latter may pose a challenge when its extruded filaments are used for 3D food printing to create 3D shapes. The higher values of cohesiveness and adhesiveness at 87% moisture content might be due to the increased flexibility and mobility of the starch chains within this range of moisture content [17], resulting in the availability of starch molecules for interaction with water and other materials. However, at a moisture content of 92%, the probability of interaction between the starch molecules and other substances decreased because of the abundance of water surrounding the starch molecules; hence, lower cohesiveness and adhesiveness values were observed. A high cohesiveness is important for the extrusion of continuous filaments. However, high adhesiveness can be more challenging during extrusion because of potential sticking to and clogging inside the nozzle, resulting in an irregular extrusion process.

### 3.3. Precise Extrusion Results

#### 3.3.1. Mass and Dimensions of the Samples After Precise Extrusion

**Figure 4:**
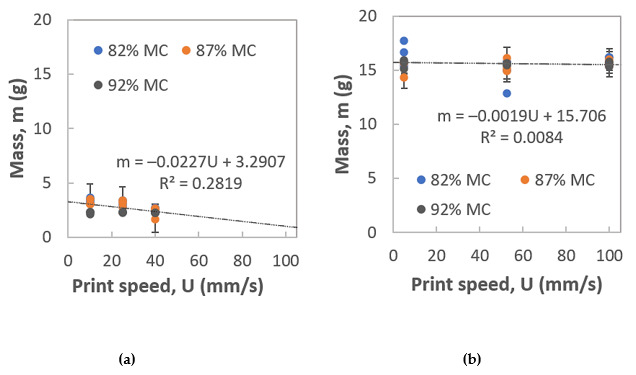
Mass of extruded filaments of sweet potato starch (SPS) sol using (**a**) a 1.5-mm nozzle and (**b**) a 4.0-mm nozzle (MC = moisture content. The dashed lines indicate the linear trends of the points).

Figure 4 shows the plots of mass versus print speed for SPS sol with different moisture contents and nozzle diameters.

Based on Figure 4, the mass values of the samples from the 4.0 mm nozzle were much higher than those from the 1.5 mm nozzle. For each nozzle, the mass decreased slightly with printing speed, although the correlation was weak. The expected 4.0 mm-to-1.5 mm ratio of the mass values is (4/1.5)^2^ = 7.11, given that the mass flow rate is directly proportional to the cross-sectional area of the nozzle, while the actual average ratio is 15.61/2.74 = 5.70. This may be attributed to the differences between the print speed (U) and the extrusion speed (V) for the 1.5 mm and 4.0 mm nozzles. In the experiment, it was the print speed that was controlled while the extrusion speed was affected by not only the print speed but also by the moisture content and the print temperature. Nonetheless, the mass of the extruded filaments was influenced by all four factors: nozzle diameter, moisture content, print temperature, and print speed.

**Figure 5:**
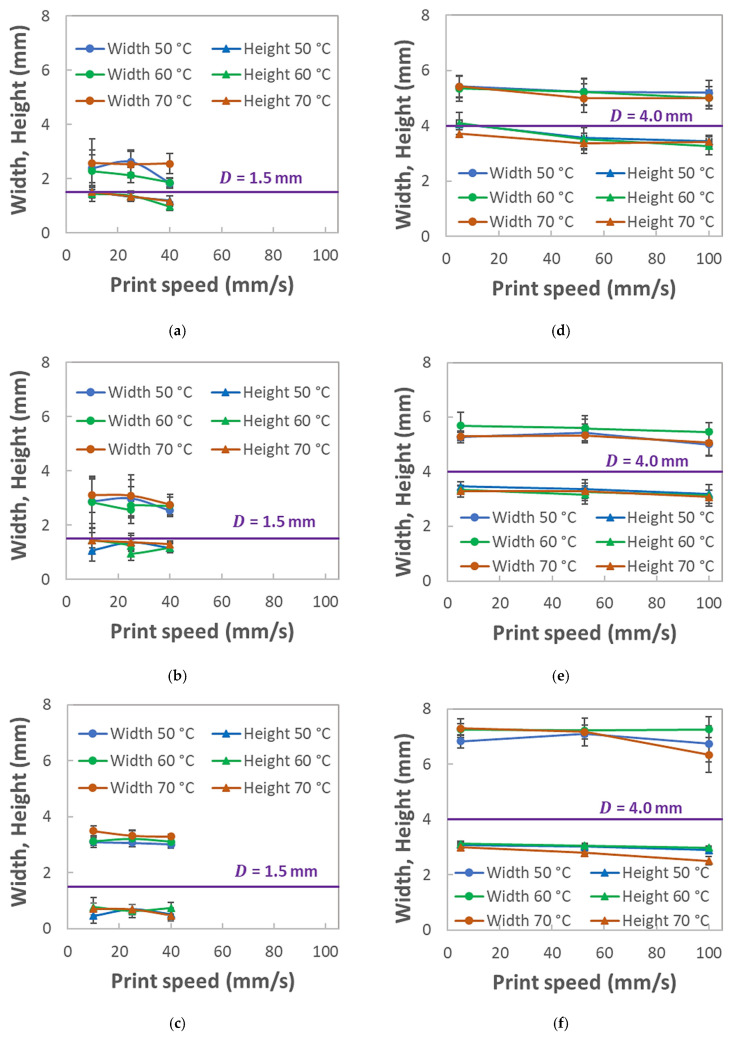
Width and height of the extruded filaments of sweet potato starch (SPS) sol using a 1.5 mm nozzle at (**a**) 82%, (**b**) 87%, and (**c**) 92% moisture content, and using a 4.0 mm nozzle at (**d**) 82%, (**e**) 87%, and (**f**) 92% moisture content. (D = nozzle diameter).

Figure 5 shows the plots of the width and height of the starch sol filaments versus the print speed at varying moisture contents (82%, 87%, and 92%) and for the nozzle diameters of 1.5 and 4.0 mm.

The width values of the samples from the 1.5 mm and 4.0 mm nozzles were greater than the nozzle diameters of 1.5 mm and 4.0 mm, respectively, which may be due to a higher extrusion speed than the print speed. The width was also observed to generally increase with the moisture content because of the spreading of the filament on the stage at higher MCs, where the starch sol was less viscous. In contrast, the height of the samples at the 1.5 mm and 4.0 mm nozzles was mostly less than the respective nozzle diameters. Significantly lower heights (p < 0.05 ) were observed at 92% MC. A possible reason for this might be the spreading of the filament on the printing stage, because the filament could not retain its circular cross-section after printing given the lower hardness, Young’s modulus (see Table 3) and apparent viscosity (see Figure S1c in the Supplementary Material) at 92% moisture content.

#### 3.3.2. Mathematical Modelling of Width and Height of the Filaments

The appropriate mathematical models were determined with the aid of Stat-Ease^®^ 360 software (trial version). The modelling process started with the selection of the type of model (i.e., linear, 2FI, quadratic, or cubic) that would give the highest value of the predicted $R^{2}$. The mathematical models were then reduced by removing the terms that were not significant based on the statistical analysis of each term in the model to provide a better prediction of the width and height given the moisture content, print temperature, and print speed. Subsequently, ANOVA for Model and ANOVA for Residual were performed to evaluate the significance of the models.

For the 1.5-mm nozzle, the width and height of the filaments were quadratic functions of the moisture content of the SPS sol, print temperature, and print speed of the 3D food printer. Based on the values of the predicted $R^{2}$ and the p-values for the ANOVA for Model and the ANOVA for Residual, the mathematical models for the width and height were used to calculate the optimum conditions. On the other hand, for the 4.0-mm nozzle, though most of the p-values showed significance for the obtained models, the ANOVA for Residual for the height model equation was “not significant” based on the p-value but was still considered because of the high value of the predicted $R^{2}$, which was 0.9344. Therefore, all the model equations were utilized for the optimization process, wherein the model and actual values were compared to verify the usefulness of the models.

#### 3.3.3. Determining and Testing the Optimum Conditions for Precise Extrusion of Sweet Potato Starch Sol

**Figure 6:**
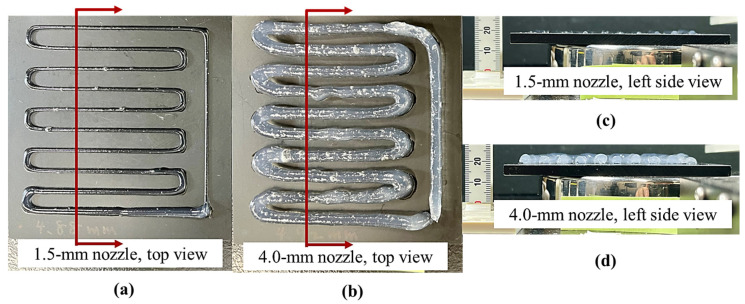
Optimized sweet potato starch sol (SPS) filaments obtained using precise extrusion: (**a**) top view with a 1.5-mm nozzle, (**b**) top view with a 4.0-mm nozzle, (**c**) left-side view with a 1.5-mm nozzle, and (**d**) left-side view with a 4.0-mm nozzle. (The red cutting plane lines show the direction of cutting of the filaments using a sharp knife to expose the cross section of the filaments to measure their width and height).

Photographs of the filaments that were precisely extruded under the optimum conditions and design patterns are shown in Figure 6.

Using the optimum printing conditions, we were able to extrude continuous filaments. However, bubble formation was inevitable during extrusion. Bubble formation may be attributed to the production of water vapor owing to the high print temperatures using the two nozzles. Bubble formation was, therefore, a limitation of this study, since the generated mathematical models did not consider this occurrence.

Table 4 presents the width and height as functions of moisture content, print temperature, and print speed for each nozzle diameter with statistical analyses for the models and residuals. Then, in Table 5, for each nozzle diameter, the optimum conditions were calculated based on the generated mathematical models, as well as the goals for optimization (see Table S1 in the Supplementary Materials), with a corresponding value of desirability. In addition, the model values of the width and height (calculated by the software) were compared with the actual (or experimental) values following the optimum conditions to test the predictive ability of the generated model equations.

**Table 4:** Reduced empirical models and statistical analysis of the models of the responses for the optimization of the precise extrusion of sweet potato starch (SPS) sol.

Nozzle Diameter (mm)	Response	Reduced Empirical Model Equation	Predicted $R^{2}$	ANOVA for Model	ANOVA for Residual
1.5	Width	$W=1.07+0.0888MC-0.2108T-0.0080U+0.0019T^{2}$	0.8264	Significant (p< 0.0001)	Not significant (p= 0.5154)
	Height	$H=-73.94+1.8265MC-0.0972U+0.0080MCU-0.0110{MC}^{2}$	0.8249	Significant (p< 0.0001)	Not significant (p= 0.8880)
4.0	Width	$W=210.44-5.0905MC+0.2847T+0.0074U-0.0002TU+0.0303{MC}^{2}-0.0023T^{2}$	0.9344	Significant (p< 0.0001)	Significant (p= 0.0303)
	Height	H=9.81−0.0667MC−0.0090T−0.0038U	0.8329	Significant (p< 0.0001)	Not significant (p= 0.4198)

*W* = filament width (mm), *H* = layer height (mm), *MC* = moisture content (% by mass, wet basis), *T* = print temperature (°C), *U* = print speed (mm/s). For the “reduced” empirical models, only the significant terms (p<0.05 for each term) were retained in the model equations. The domains of the models are 82% < MC < 92% and 50 °C < T < 70 °C for both nozzle diameters, and 10 mm/s < U < 40 mm/s for the 1.5 mm nozzle and 5 mm/s < U < 100 mm/s for the 4.0 mm nozzle. For ANOVA for Model and ANOVA for Residual, the result is “significant” if p<0.05 and “not significant” if p>0.05.

**Table 5:** Optimum conditions for the precise extrusion of sweet potato starch (SPS) sol for each nozzle diameter.

Nozzle Diameter (mm)	Moisture Content (%)	Print Temperature (°C)	Print Speed (mm/s)	Desirability	Width (mm)		Height (mm)	
					Model	Actual	Model	Actual
1.5	82	56.6	10	0.842	2.31 ± 0.16 ^c^	2.53 ± 0.65 ^c^	1.49 ± 0.13 ^d^	1.08 ± 0.44 ^d^
4	82.3	50	5	0.911	5.18 ± 0.18 ^a^	5.56 ± 0.30 ^a^	3.86 ± 0.12 ^b^	3.95 ± 1.21 ^b^

Means with the same superscripts are not significantly different at p> 0.05, HSD.

For the 1.5 mm nozzle, the optimum conditions were 82% moisture content, a print temperature of 56.6 °C (although 57 °C was used instead in the 3D food printer because the printer settings only allow a multiple of 1 °C), and a print speed of 10 mm/s, with a desirability of 0.842. In contrast, for the 4.0 mm nozzle, the optimum conditions were 82.3% moisture content, a print temperature of 50 °C, and a print speed of 5 mm/s, with a desirability of 0.911. These desirability values were relatively high given that there were only two responses (width and height) in the RSM process.

Based on the statistical analysis, there was no significant difference between the model and actual (experimental) values, which may be attributed to the high desirability values. Thus, the model equations obtained using the software were useful during the optimization process.

Given that the optimum conditions were found to be useful to create filaments that are smooth, continuous, and made with high precision, the mechanism by which such filaments were produced was studied.

### 3.4. Mechanism Analysis

The precise extrusion behavior of the SPS sol filaments was controlled mainly by the material’s viscoelastic response to different printing conditions. The moisture content and the print temperature influenced the balance between viscous flow and elastic recovery, and consequently the filament width and height (as discussed in Section 3.3). Increasing the moisture content weakened the intermolecular starch interactions and reduced the apparent viscosity, causing greater flow upon deposition and resulting in wider and flattened filaments. However, lower moisture levels increased the structural resistance to deformation, resulting in better shape retention of the extruded filaments. Furthermore, elevated print temperatures enhanced the mobility of the starch molecules and reduced the flow resistance during extrusion but also increased the spreading of the filaments upon deposition because of weaker elastic recovery. Therefore, the dimensional stability and the precision of the deposited filaments were determined by the interaction between viscous deformation during extrusion and elastic recovery after deposition.

There are four phases of screw-type 3D food printing, as suggested in [23], which can be applied to the precise extrusion of SPS sol filaments.

#### 3.4.1. Phase I: Ability to Transition to a Viscous Body

In this phase, the SPS sol was initially in a static state, so the viscoelastic food material had predominantly solid-like properties. During extrusion, the material should be liquid-like to achieve better extrudability [23]. Hence, to determine which property, solid-like or liquid-like, was more dominant during extrusion, the loss factor during extrusion was analyzed given the elastic modulus ($G^{\prime }$) and viscous modulus ($G^{\prime \prime }$) corresponding to the shear strain during extrusion ($\gamma_{E}$).

(2) $$\tan\delta_{\gamma E}=\frac{{G^{\prime \prime }}_{\gamma E}}{{G^{\prime }}_{\gamma E}}$$

The shear strain during extrusion was calculated based on the length ($L_{n}$) and the inner diameter (D) of the nozzle.

(3) $$\gamma_{E}=\frac{2L_{n}}{D}$$

**Figure 7:**
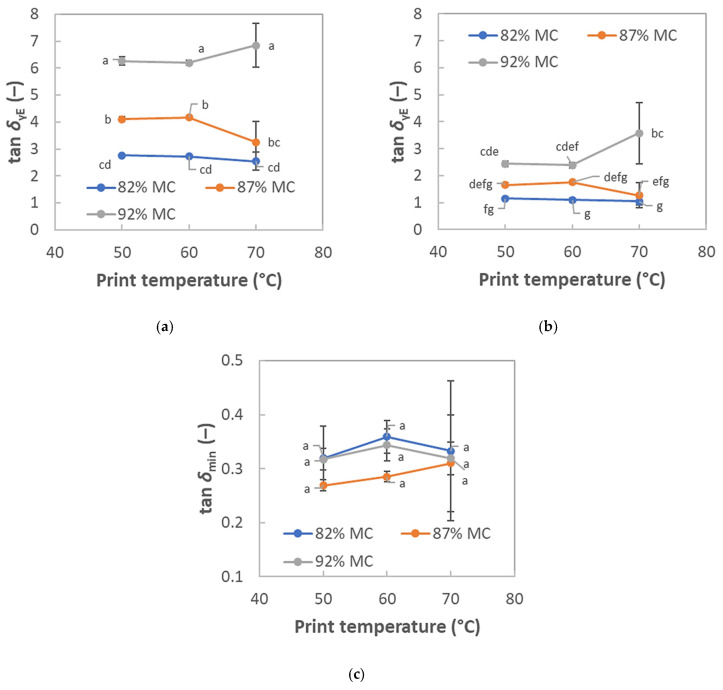
Loss factor during extrusion of sweet potato starch (SPS) sol using (**a**) 1.5 mm nozzle where $\gamma_{E}$ = 32, (**b**) 4.0 mm nozzle where $\gamma_{E}$ = 12, and (**c**) just after extrusion or at minimum strain. ($\gamma_{E}$ = shear strain during extrusion. Points with the same labels are not significantly different at p> 0.05, HSD).

Given that the nozzles used in the study were 1.5 mm and 4.0 mm, the respective shear strains during extrusion were 32 and 12. Figure 3 was then used to determine the corresponding $G^{\prime }$ and $G^{\prime \prime }$, and then $\tan\delta_{\gamma E}$ was calculated accordingly and is shown in Figure 7.

The range of values of 1.05–6.84 for the loss factor was observed for both the nozzles, such that all the values were greater than 1, suggesting a more liquid-like viscoelastic material during extrusion. An increase in moisture content tends to make the viscoelastic material more liquid-like. However, for each moisture content, the print temperature had no significant effect on the loss factor. Moreover, the 1.5-mm nozzle produced relatively higher values of loss factor, probably due to a greater value of $\gamma_{E}$ wherein the loss factor was greater when the strain was higher.

#### 3.4.2. Phase II: Ability to Be Extruded

**Figure 8:**
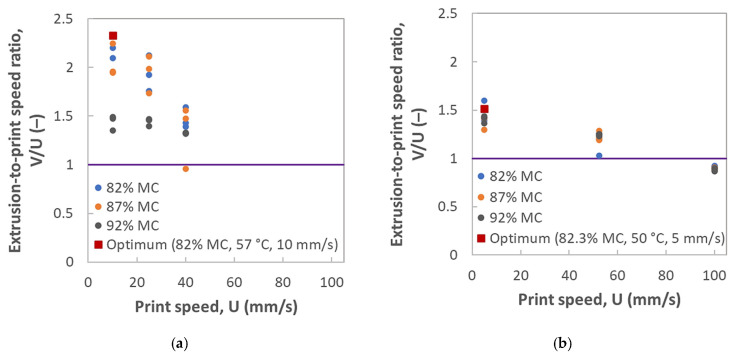
Extrusion-to-print speed ratio (V/U) of sweet potato starch (SPS) sol using (**a**) 1.5-mm and (**b**) 4.0-mm nozzles. (Purple lines indicate an ideal extrusion-to-print speed ratio of 1).

In this phase, the ability of the food material to be extruded from the nozzle is evaluated in terms of the extrusion-to-print speed ratio. If the ratio of the extrusion speed to print speed (V/U) is close to one, a smooth and continuous filament can be printed, and the width of the filament can be as close as possible to the nozzle diameter [28]. In Figure 8, the extrusion speed (V) was estimated given the total mass (m) of the extruded filaments, the nozzle diameter (D), the density of the food material (ρ), assumed to be 0.001 g/mm^3^, and the total printing time (t).

(4) $$V=\frac{4m}{\pi D^{2}\rho t}$$

For both the nozzles, most of the samples had an extrusion-to-print speed ratio slightly greater than one, with some approaching two. The other samples had a ratio of less than one, with 0.87 as the lowest value, but still close to unity. For example, a value slightly greater than one indicates that the extrusion speed is slightly greater than the print speed, resulting in a filament with a width slightly greater than the nozzle diameter, in addition to the spreading of the filament as a viscous body. Nevertheless, the filaments were successfully extruded using the 1.5 mm and 4.0 mm nozzles. Moreover, for the optimum conditions for each nozzle diameter of 1.5 mm and 4.0 mm, the respective ratios are 2.33 and 1.52, which may not be that close to one, but nonetheless, the optimum conditions included not only the extrusion and print speeds but also the print temperature and moisture content.

#### 3.4.3. Phase III: Ability to Return from Liquid-like to Solid-like

After extrusion, the filaments, as viscoelastic food materials, returned to a more elastic (solid-like) than viscous (liquid-like) state. Shear strain on the filaments may still be present, but at a sufficiently low value. In the experiment using the rheometer, the lowest strain value in the data was 0.0003. Hence, the loss factor at the minimum strain ($\tan\delta_{min}$) was evaluated (see Figure 7). Based on the statistical analysis, the values of the loss factor did not differ significantly from each other. Nevertheless, all these values are much less than one, or approximately 0.3. Therefore, the SPS sol can return to a more elastic state immediately after extrusion.

#### 3.4.4. Phase IV: Ability to Retain Shape

In the previous Section, because the values of the loss factor immediately following extrusion (or at minimum strain) were considerably lower than one, it can be said that the elastic component of the viscoelastic food material was predominant after extrusion. Thus, Young’s modulus, which assumes elastic material, was considered (Table 3). The Young’s modulus measures the linear stiffness of a food material under tension or compression. It was calculated by taking the slope of the linear elastic region in the stress–strain curve produced by the texture analyzer. It is important to assess the shape stability of 3D-printed foods, which is affected by their hardness and adhesiveness [10,23]. A higher Young’s modulus indicates greater tensile or compressive force, which causes deformation (in terms of strain) through tension or compression. In this study, the stiffness of the extruded filaments was important because several layers of filaments were printed to form the 3D shapes.

During the experiment, points with high linearity were observed at strain values of 4–10%, similar to the studies by [23,29]. From the above Figure, the Young’s modulus values were observed at 1090–4382 Pa at varying moisture levels and print temperatures. This range is smaller than that of pumpkin paste (10,160–141,560 Pa) [23] and nixtamalized corn paste (1538–146,729 Pa) [24], probably because the mentioned pastes were from flour that contained crude protein and fiber that contributed to the hardness of the pastes, while the solids from SPS sol were from starch and minute amount of impurities only. In addition, the extrusion process for the sol was conducted at a relatively high temperature compared to the pastes that were extruded at room temperature, and the hot material was expected to be softer. Nevertheless, when the hot sol cools at room temperature, it turns into a gel, which is a harder material that has a greater ability to retain its shape after extrusion.

## 4. Conclusions

Unlike food materials such as pastes that are made of flour and water that can be extruded at room temperature, starch should be prepared as a liquid-like sol that can be extruded through a nozzle to form filaments. It should be maintained in its sol form until the end of the extrusion process, where it can turn into a solid-like gel that is more capable of retaining its shape. Based on its rheological and mechanical properties, SPS sol is a suitable food material for the precise extrusion of filaments. However, the optimization of the printer settings (i.e., print temperature and print speed) as well as the preparation of the material (i.e., starch or water content, and sol instead of gel form) is necessary for a successful printing.

## Figures and Tables

**Table 1:** Complete factorial design (CFD) for optimizing printing conditions for the precise extrusion of sweet potato starch (SPS) sol using 1.5- and 4.0-mm nozzles.

Run	Factors			
	Moisture Content, MC (%)	Print Temperature, T (°C)	Print Speed, U (mm/s)	
			1.5 mm Nozzle	4.0 mm Nozzle
1	87	60	40	100
2	87	50	10	5
3	87	70	10	5
4	87	60	10	5
5	87	60	25	52.5
6	87	50	25	52.5
7	87	50	25	52.5
8	92	70	10	5
9	82	50	40	100
10	92	70	25	52.5
11	92	50	40	100
12	92	70	10	5
13	82	50	10	5
14	87	50	40	100
15	92	50	25	52.5
16	92	70	40	100
17	82	60	40	100
18	87	70	25	52.5
19	82	50	25	52.5
20	92	60	10	5
21	87	70	40	100
22	82	70	10	5
23	92	60	25	52.5
24	82	60	10	5
25	92	60	40	100
26	82	60	25	52.5
27	82	70	25	52.5
28	82	70	40	100

**Table 2:** Herschel–Bulkley parameters for sweet potato starch (SPS) sol.

Moisture Content, MC (%)	Print Temperature, T (°C)	Yield Stress, $\tau_{0}$ (Pa)	Consistency Coefficient, K (Pa·s^n^)	Flow Index, n (−)
82	50	713.8 ± 130.3 ^a^	72.2 ± 7.45 ^a^	0.59 ± 0.02 ^bc^
	60	353.0 ± 120.5 ^abc^	40.0 ± 6.1 ^b^	0.62 ± 0.03 ^bc^
	70	222.0 ± 3.1 ^bcd^	28.4 ± 4.1 ^bc^	0.64 ± 0.04 ^abc^
87	50	568.7 ± 363.5 ^ab^	24.0 ± 2.6 ^cd^	0.70 ± 0.01 ^abc^
	60	361.2 ± 171.0 ^bcd^	18.7 ± 5.6 ^cde^	0.67 ± 0.05 ^abc^
	70	190.1 ± 61.4 ^cd^	11.0 ± 2.2 ^def^	0.69 ± 0.07 ^abc^
92	50	118.0 ± 3.7 ^cd^	5.4 ± 1.4 ^ef^	0.79 ± 0.05 ^a^
	60	114.4 ± 2.1 ^d^	5.0 ± 0.6 ^ef^	0.75 ± 0.01 ^abc^
	70	70.3 ± 20.2 ^bcd^	4.6 ± 7.7 ^f^	0.55 ± 0.13 ^c^

Means with the same superscripts are not significantly different at p> 0.05, HSD.

**Table 3:** Effects of moisture content and print temperature on the mechanical properties of sweet potato starch (SPS) sol.

Moisture Content, MC (%)	Print Temperature, T (°C)	Hardness (Pa)	Cohesiveness (–)	Adhesiveness (J/m^3^)	Young’s Modulus (Pa)
82	50	2571 ± 458 ^a^	0.85 ± 0.10 ^a^	3000 ± 1626 ^bcd^	4052 ± 196 ^ab^
	60	2911 ± 684 ^a^	0.89 ± 0.14 ^a^	2950 ± 1077 ^bcd^	4382 ± 786 ^a^
	70	2647 ± 285 ^a^	0.96 ± 0.02 ^a^	4573 ± 1641 ^abc^	3844 ± 518 ^ab^
87	50	1096 ± 173 ^bc^	0.96 ± 0.04 ^a^	4422 ± 967 ^abc^	2440 ± 398 ^c^
	60	1021 ± 196 ^bc^	0.99 ± 0.09 ^a^	6143 ± 550 ^a^	2415 ± 218 ^c^
	70	1664 ± 173 ^b^	0.95 ± 0.14 ^a^	5788 ± 597 ^ab^	3127 ± 206 ^bc^
92	50	794 ± 113 ^bc^	0.83 ± 0.06 ^a^	947 ± 146 ^d^	1317 ± 176 ^d^
	60	718 ± 131 ^c^	0.77 ± 0.06 ^ab^	1318 ± 444 ^d^	1204 ± 46 ^d^
	70	832 ± 65 ^bc^	0.44 ± 0.07 ^b^	2192 ± 756 ^cd^	1090 ± 211 ^d^

The texture meter settings were as follows: mass of 28 g sample in a stainless-steel container (diameter of 48 mm and a height of 15 mm), 16 mm diameter plunger, compression speed of 5 mm/s, clearance of 5 mm, and two measurement cycles. Means with the same superscripts are not significantly different at p> 0.05, HSD.

## Data Availability

The original contributions of this study are included in the Article and the Supplementary Materials. Further inquiries can be directed to the corresponding author.

## Supplementary Materials

The following supporting information can be downloaded at: https://www.mdpi.com/article/10.3390/foods15122204/s1, Figure S1: Effect of shear rate on the apparent viscosity of sweet potato starch sol at (a) 82%, (b) 87%, and (c) 92% moisture content; Table S1: Goals for optimizing the precise extrusion of sweet potato starch sol; Table S2: Confirmation of optimum conditions for precise extrusion of sweet potato starch sol.

## Abbreviations

The following abbreviations are used in this manuscript:

SPS	sweet potato starch
MC	moisture content
DOE	design of experiment
CFD	complete factorial design
