# Supplementary material for: Precise Extrusion of Sweet Potato (Ipomoea batatas L.) Starch Sol Filaments: Printability Analysis and Process Optimization
Source: Foods. 2026 Jun 18;15(12):2204. doi: 10.3390/foods15122204 (PMC13298275; doi:10.3390/foods15122204)
Supplement: Supplementary file 1 [file foods-15-02204-s001.zip › foods-4258413-supplementary.pdf]

Supplementary material for

Precise Extrusion of Sweet Potato (*Ipomoea batatas* L.) Starch Sol Filaments: Printability Analysis and Process Optimization

Al Kaxier G. Ancheta[1,2], Hiroyuki Kozu[3], Takumi Umeda[3], Marcos A. Neves[4] and Isao Kobayashi[3,5]*

[1] Graduate School of Science and Technology, University of Tsukuba, Tsukuba 305-8577, Japan

[2] Department of Engineering Science, College of Engineering and Agro-industrial Technology, University of the Philippines Los Baños, Los Banos 4031, Philippines

[3] Institute of Food Research, National Agriculture and Food Research Organization (NARO), Tsukuba 305-8642, Japan

[4] Institute of Life and Environmental Sciences, University of Tsukuba, Tsukuba 305-8577, Japan

[5] School of Integrative and Global Majors, University of Tsukuba, Tsukuba 305-8577, Japan

*Correspondence: kobayashi.isao697@naro.go.jp

Supplementary Material

Apparent viscosity of sweet potato starch sol

Figure S1 presents the apparent viscosity of the same food material at varying moisture contents and print temperatures.

(a)

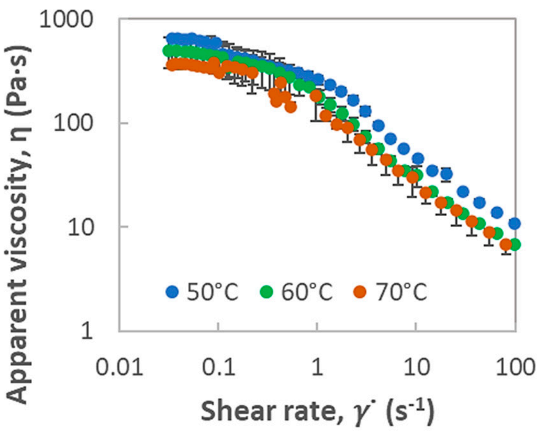

(b)

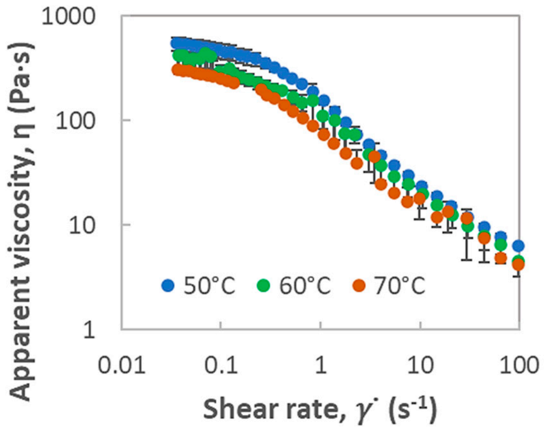

(c)

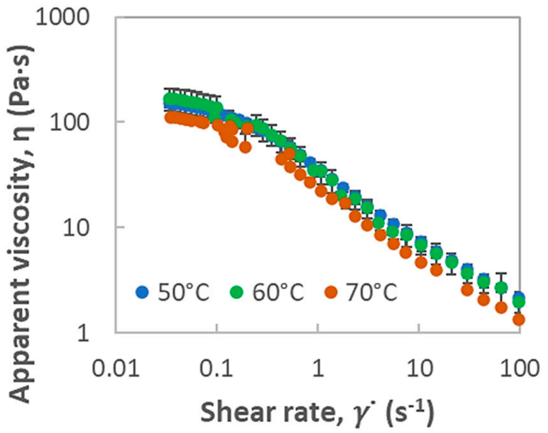

Figure S1. Effect of shear rate on the apparent viscosity of sweet potato starch sol at **(a)** 82, **(b)** 87, and **(c)** 92% moisture content.

Given that apparent viscosity is defined as the ratio of shear stress to shear rate, the decreasing viscosity values clearly demonstrate shear-thinning behavior, regardless of the print temperature.

Goals for optimization of precise extrusion of sweet potato starch sol

The ranges of values for the width and height for nozzle diameters of 1.5 mm and 4.0 mm are summarized in Table S1 to help in the careful decision-making for the optimization goals.

Table S1. Goals for optimizing the precise extrusion of sweet potato starch sol.

Nozzle diameter (mm)	Response	Range of Values* (mm)	Goal
1.5	Width, W	1.84–3.5	Minimize
	Height, H	0.46–1.53	target (1.5 mm)
4	Width, W	5–7.3	Minimize
	Height, H	2.51–4.09	target (4.0 mm)

*Obtained from experimental data

For the 1.5-mm nozzle, given that the range of values of width (1.84–3.5) obtained from the experiment were all greater than the ideal width of 1.5 mm, the goal was to minimize the value to make it as close as possible to the ideal value. For the height, as the ideal height of 1.5 mm was within the range of values (0.46–1.53), the goal was to neither minimize nor maximize, but to hit the target, which is 1.5 mm. Similar observations (for the range of values of width and height) and goals were for the 4.0-mm nozzle.

Confirmation of optimum conditions

To supplement the results from Table 5 (in the main text), additional results are shown in Table S2.

Table S2. Confirmation of optimum conditions for precise extrusion of sweet potato starch sol.

Nozzle diameter (mm)	Response	Model value (mm)	Actual value (mm)	Relative error (%)	95% Prediction interval
1.5	Width	2.31 ± 0.16 ^{c}	2.53 ± 0.65 ^{c}	9.52	2.04–2.58
	Height	1.49 ± 0.13 ^{d}	1.08 ± 0.44 ^{d}	27.52	1.26–1.72
4.0	Width	5.18 ± 0.18 ^{a}	5.56 ± 0.30 ^{a}	7.34	4.85–5.52
	Height	3.86 ± 0.12 ^{b}	3.95 ± 1.21 ^{b}	2.33	3.64–4.07

Note: Confidence level of 95%

% Relative error = | Actual value - Model value | / Model value × 100

Prediction interval expressed as a range of values showing the lowest and highest values

In the main text, Table 5 shows the statistical analysis comparing the model and actual values in terms of mean ± standard deviation. The model and actual values are not significantly different at $p > 0.05$ for each nozzle diameter and response. However, in Table S2, a large deviation from the model was observed for the actual value for height at 1.5-mm nozzle. In terms of prediction interval at 95% confidence level, most of the actual values are within the prediction intervals. While the confidence interval estimates the likely range of the mean result, the prediction interval is much wider because it accounts for the inevitable variation of individual single runs. The prediction interval can also be used to manage expectations for future run of 3D food printing using the optimum conditions. Nonetheless, the generated models in Table 4 and the optimum conditions in Table 5 of the main text were found to be useful for precise extrusion of the filaments.
